# Structure and establishment of the German Cochlear Implant Registry (DCIR)

**DOI:** 10.1007/s00106-023-01310-0

**Published:** 2023-07-18

**Authors:** T. Stöver, S. K. Plontke, O. Guntinas-Lichius, H-J. Welkoborsky, T. Zahnert, K. W. Delank, T. Deitmer, D. Esser, A. Dietz, A. Wienke, A. Loth, S. Dazert

**Affiliations:** 1 Department of Otorhinolaryngology, University Hospital Frankfurt, Theodor-Stern-Kai 7, 60590 Frankfurt, Germany; 2 Department of Otorhinolaryngology, Head and Neck Surgery, University Medical Center Halle, Halle (Saale), Germany; 3 Department of Otorhinolaryngology, Jena University Hospital, Jena, Germany; 4 Hospital for Otorhinolaryngology, Klinikum Nordstadt, Hanover, Germany; 5 Department of Otorhinolaryngology, Dresden University Hospital, Dresden, Germany; 6 Department of Otorhinolaryngology, Ludwigshafen Hospital, Ludwigshafen, Germany; 7 Department of Otorhinolaryngology, Leipzig University Hospital, Leipzig, Germany; 8 Law office WBK, lawyer specialist medical law, Cologne, Germany; 9 Department of Otorhinolaryngology, University Hospital (St. Elisabeth Hospital), Bochum, Germany

**Keywords:** Rehabilitation, Implantable neurostimulators, Prostheses and implants, Certification, Quality control

## Abstract

Hearing rehabilitation of patients with severe hearing loss or deafness using cochlear implants (CI) is a very successful but also complex and lifelong process that requires high quality standards for structure, process, and results. Medical registries represent an ideal tool for conducting quality control relevant to care while at the same time collecting scientific data. Therefore, at the initiative of the Executive Committee of the German Society of Oto-Rhino-Laryngology, Head and Neck Surgery (DGHNO-KHC), a Germany-wide CI registry (the German Cochlear Implant Register, DCIR) was to be established. The following goals were to be achieved: 1) legal and contractual basis for the register; 2) definition of the register contents; 3) development of evaluation standards (hospital-specific and national annual reports); 4) development of a logo; 5) practical operation of the registry. After defining a catalog of services that defines the content, structure, and operation of the DCIR, a registry operator with audiological expertise was sought. After considering various offers, the registry was technically implemented in cooperation with the provider INNOFORCE (Ruggell, Liechtenstein) as registry operator. This also included the realization of an interface for data transfer from previously existing databases and development of a data protection concept for productive operation of the DCIR under the scientific leadership of the DGHNO-KHC Executive Committee. Since January 2022, it has been possible for participating hospitals to enter pseudonymized data into the DCIR. To date, 75 hospitals in Germany have contractually agreed to participate in the registry. During the first 15 months, data from over 2500 implants in over 2000 patients were registered in the DCIR. The work presented here describes the structuring, development, and successful establishment of the DCIR. Introduction of the DCIR represents an important milestone of future scientifically based quality control in CI care. The registry presented here can therefore be considered as an example for other areas of medical care and thus also sets an international standard.

**Supplementary Information:**

The online version of this article (10.1007/s00106-023-01310-0) contains supplemental material: Data blocks CI registry.

## Quality control

Cochlear implant (CI) treatment is a very successful but also complex and lifelong process for patients who suffer from profound hearing loss or deafness [1]. The medical–scientific basis of CI care in Germany is highly standardized and defined in detail by an AWMF guideline (*Arbeitsgemeinschaft der Medizinisch-Wissenschaftlichen Fachgesellschaften* = Working Group of the Medical–Scientific Societies, register no. 017-071; [2]). In an elaborate consensus process, the current third version of the guideline was adopted in 2020 under the leadership of the German Society of Otorhinolaryngology, Head and Neck Surgery e. V. (DGHNO-KHC) with other medical societies that are also important for CI care, the German Society of Phoniatrics and Pediatric Audiology (DGPP) and the German Audiological Society (DGA). The current guideline describes not only the medical and scientific standard of diagnostics, surgery, and postoperative care currently applicable in Germany, but also the necessary lifelong aftercare process. This document is thus a milestone in the quality control of CI care not only in Germany, but worldwide. For the first time, essential aspects of structural quality, process quality, and outcome quality for CI care have been defined here. Based on this guideline, a practical recommendation for the implementation of the guideline content was developed by the Executive Committee of the DGNHO-KHC and also jointly consented upon by the relevant medical societies (DGHNO-KHC, DGPP, DGA; CI white paper of the DGHNO-KHC; [3]).

The quality control of a complex and lifelong process, such as CI therapy, represents a major challenge. The ultimate responsibility for the lifelong CI care process undoubtedly lies with the institution providing cochlear implants (CIVE). Usually, this is a main department for otorhinolaryngology, head and neck surgery [2]. This is based on medical responsibility (e.g., medical indication, surgical implantation, coordination, and responsibility for the overall process). In addition, there are legal requirements (“Medical Devices Operator Ordinance”—MPBetreibV), according to which the CIVE is to be regarded as the “operator of the implant” [4].

## Significance of medical registries

The use of medical registries is an effective tool for quality control in this context and at the same time for collecting scientific data that can also form the basis for future guideline developments. This is especially true if these clinical data are not only collected on a multicenter basis, but on a nationwide basis. For a number of medical implants or diseases, medical registries have already been operated very successfully for many years. The trauma registry [5] and the endoprosthesis registry [6] should be mentioned here.

Although CI care has been available in Germany since the end of the 1980s, there are still insufficient national data on the number of patients treated with CIs, complications, manufacturer-independent recording of implant safety, long-term stability of hearing improvement with CIs, and long-term effects on quality of life. The *Implantateregistergesetz—IRegG* [7] provides for mandatory documentation of CIs in the future. However, the practical implementation of this law for CIs is currently not exactly predictable with regard to a concrete date.

In the development of both the CI guideline and the CI white paper, it became clear that the quality criteria developed in each case can only represent the currently known scientific status. This led to the conclusion that only the establishment of a national register would allow to address scientifically relevant questions as well as to develop future quality parameters. In this respect, the development of a Germany-wide CI registry represents a consistent continuation of the future-oriented continuous development of the CI guideline and the CI white paper and thus of further quality control of CI care.

The basic content of the CI registry presented here has already been developed by the DGHNO-KHC for the second version of the CI white paper in May 2021 [3]. Therefore, on the initiative of Executive Committee of the DGHNO-KHC, a Germany-wide CI registry (German Cochlear Implant Registry = DCIR) should be established on the basis of the AWMF CI guideline and the CI white paper. For this purpose, the following goals should be achieved:

1. Development of the legal and contractual basis for the establishment and operation of a clinical registry under the scientific direction of the DGHNO-KHC
2. Definition of the register content based on the current CI guideline and the CI white paper
3. Development of an evaluation standard (hospital-specific and national annual reports)
4. Development of a DCIR logo
5. Start of data entry and practical operation of the DCIR

## Material and methods

### Scientific basis of the DCIR

The process of hearing rehabilitation with a CI in Germany is described in detail by the AWMF CI guideline [2] and takes into account the structural quality, the process quality, and the outcome quality of the complete care process. This guideline was developed with consensus of the medical–scientific societies relevant for CI treatment, the DGHNO-KHC, the DGPP, and the DGA. This guideline thus represents a milestone in the standardization of CI treatment in Germany. On this basis, the consented practical implementation recommendations were developed under the leadership of the DGHNO-KHC and published as the CI white paper in 2021 [3]. The CI white paper already described the main features of a Germany-wide CI registry, whose practical implementation as DCIR [8] is described in this paper.

### Decision-making process for the establishment of the DCIR

In parallel to the development of the CI guideline and the CI white paper for structuring the CI care process, an independent certification process for quality assurance of CI care was subsequently introduced in Germany [9]. As early as in 2016, after intensive discussion, the Executive Committee of the DGHNO-KHC made the decision to participate in the further development of quality control of CI care in a scientifically oriented manner. For this purpose, the establishment of a national CI registry was also considered essential. After completing the necessary technical preparatory work (CI guideline and CI white paper), the decision of the Executive Committee of the DGHNO-KHC to cooperate with an external registry operator was finally made in November 2021. Various potential providers were considered, and a registry operator with great audiological expertise was sought. The DGHNO-KHC board decided to implement the DCIR in cooperation with INNOFORCE (Ruggell, Liechtenstein) as the registry operator. The implementation of the DCIR was carried out under the scientific direction of the DGHNO-KHC board.

### Organizational structure and legal relationships

The Executive Committee of the DGHNO-KHC developed a service catalog on the basis of which the content, structure, and operation of the DCIR by the registry operator were determined. The criteria essentially included the technical implementation of a registry database including an application programming interface (API), an interface for the transfer of data from databases already existing at the hospitals, the development of a data protection concept, and the practical operation of the DCIR. The processing of the pseudonymized data for the preparation of an annual report for each participating hospital and the preparation of a national annual report for the DGHNO-KHC are also among the agreed tasks of the registry operator.

**Fig. 1:**
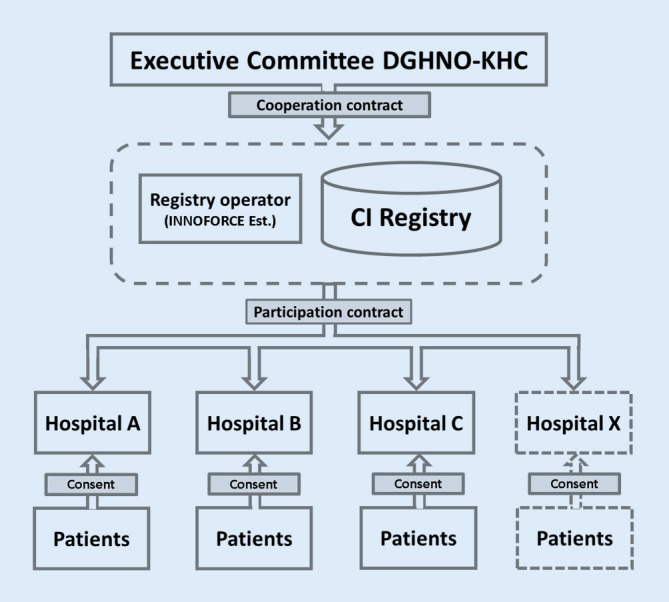
Schematic representation of the organizational and legal relationship between the DGHNO-KHC, the registry operator (INNOFORCE, Ruggell, Liechtenstein), the participating hospitals, and the patients

For this purpose, the registry operator, as the responsible party under data protection law, concludes a participation agreement in the DCIR with each of the interested hospitals. The respective hospital receives the annual report on the data entered in each calendar year. Only pseudonymized data are transmitted to the DCIR. The tasks of the participating hospitals include informing the patients whose data are registered about the objectives and the data protection concept, as well as obtaining and documenting individual patient consent for data transfer to the DCIR (Fig. 1).

There is no direct contractual legal relationship between the operator of the DCIR and patients. Likewise, there is no direct legal relationship between the participating hospitals and the Executive Committee of the DGHNO-KHC with regard to the DCIR. The scientific management of the DCIR lies with the Executive Committee of the DGHNO-KHC, as do the rights of use of the anonymized national data.

### Data protection concept

The collection of clinical data from patient care for the DCIR requires consent from each patient, even when using pseudonymized data. This consent therefore had to be obtained from the participating hospital for each patient whose data were to be registered in the DCIR. For this purpose, a model consent form was developed and made available to the participating hospitals.

Data transfer from a participating hospital to the DCIR should be performed exclusively on the basis of pseudonymized data. The identification of individual patients or their data after transfer to the DCIR is therefore not possible for the registry operator or for the Executive Committee of the DGHNO-KHC.

**Fig. 2:**
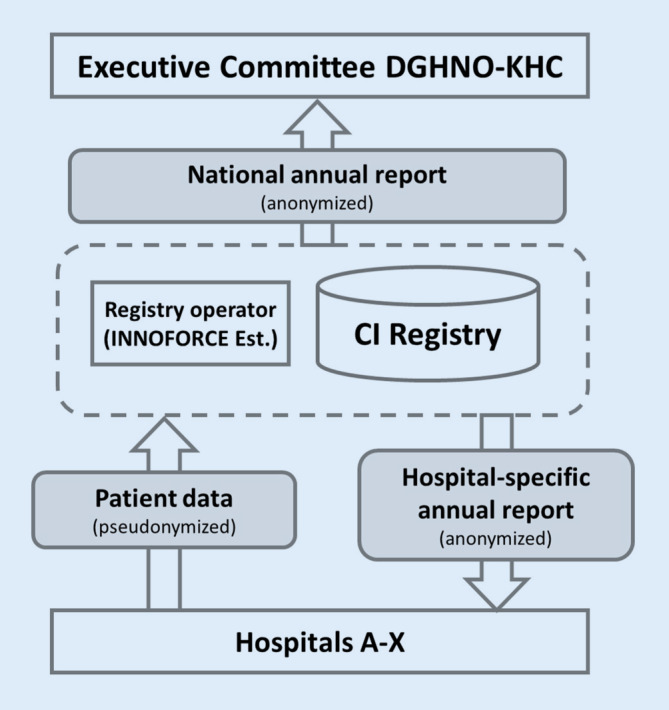
Schematic representation of the data flow for the operation of the registry as well as the resulting annual reports to the participating hospitals and the DGHNO-KHC

Each participating hospital should receive the data it has entered into the registry as an anonymized annual report. The annual report is thus a benchmark with which to compare the respective hospital data (e.g., number of complications) with the national overall data of the DCIR. The DGHNO-KHC Executive Committee receives an anonymized national annual report of all data without allowing conclusions to be drawn about individual hospitals or individual patients (Fig. 2). The data protection concept presented was reviewed both legally and by the data protection officers of the respective hospitals before the DCIR went into operation.

### Technical implementation of data entry into the CI registry

**Fig. 3:**
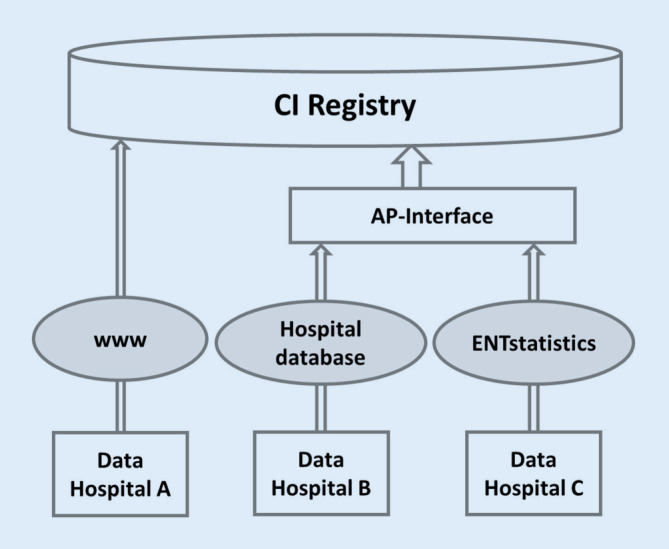
Presentation of the available data entry options for the CI registry: Browser-based data entry using the example of hospital A. Data transfer from an existing hospital database (hospital B) via the application programming (*AP*) interface or the use of ENTstatistics (hospital C). Both the use of a hospital database and ENTstatistics enable automated data import from audiometric workstations

During the conception phase of the DCIR, a very heterogeneous starting situation became apparent with regard to the databases and documentation systems used for quality control of CI care in the hospitals. A solution that could be implemented for all hospitals willing to participate therefore had to take into account the different initial situations on the one hand and ensure homogeneous data quality of the DCIR on the other. Consequently, various technical access options for data transfer were developed and offered to the hospitals individually for use. These included (1) Internet-based data entry, (2) the use of an already existing database, or (3) the establishment of a database of the registry operator (Fig. 3).

#### Internet-based data entry

For hospitals that either have not operated their own IT system for the documentation of CI-related data so far or only provide care for a few CI cases per year, the possibility of a direct Internet-based data entry for the DCIR should be possible. For this purpose, the registry operator has developed an Internet-based registry access that enables online “manual” entry of registry data. This access thus allows a hospital to participate in the DCIR even without further technical requirements, such as the establishment of a separate hospital database.

#### Transfer of data from an existing hospital database

A large number of hospitals participating in the DCIR already operate their own hospital databases or documentation systems for quality assurance of CI care. Most of these local databases can connect peripheral devices (e.g., audiometers) and import results. In order to avoid duplicate data collection, it was necessary to create the possibility to transfer the locally collected CI therapy data to the registry. For this purpose, an API has been provided by the registry operator since fall 2022.

#### Use of the database of the registry operator

The registry operator (INNOFORCE, Ruggell, Liechtenstein) has an ENT database (ENTstatistics; [10]). This system is used by many hospitals in Germany to document and statistically evaluate otologic, rhinologic, laryngologic, and tumor findings. In particular, ENTstatistics offers interfaces for the integration of peripheral endpoints. The system supports the documentation of therapy data required for the DCIR as well as the subsequent transfer to the DCIR.

### Content of the DCIR: data blocks

The DCIR is primarily oriented toward the documentation of the implant or implantation. Thus, only patients who have actually received an implant will be included in the registry. The registry is purely prospective, so that implants and implantations could only be registered from the time the registry started operating (January 2022). The registry system is therefore based on the recording and documentation of parameters relevant for the assessment of implant function. These are divided into ten so-called data blocks, which are based on the current AWMF CI guideline [2]. These include in detail: baseline data, preoperative audiometry, preoperative hearing history, implant, surgery, CI-related complications, CI use and rehabilitation progress, postoperative audiometry, hearing/language development (children), and quality of life. In addition, however, the data blocks also include the documentation of guideline-compliant CI care. This treatment process includes the preoperative phase, the operative phase, the basic therapy, the follow-up therapy, as well as the lifelong aftercare. The definition and content of the data blocks have already been integrated into the current version of the CI white paper of the DGHNO-KHC [3]. An overview of the data blocks and their content can be found in Table 1 and the complete list of all collected registry parameters in the attached supplement.

**Table 1:** Overview of the ten data blocks used in the DCIR including their subcategories

*1. Basic data*	*6. CI-related complication*
ID (code) provision facility	Electrode misalignment in need of revision
Patient ID pseudonym	Facial palsy
Date of birth, date of death; gender; mother tongue	Hospital admission due to CI-related complication
*2. Preoperative audiometry*	Meningitis related to CI treatment
Pure tone audiogram	Death in relation to CI treatment
Speech test results; sentence test results	*7. CI use and rehabilitation*
Objective measurement (OAE, ABR, ASSR)	Implant function
*3. Preoperative hearing history*	Useful life
Time of hearing loss	Current rehabilitation status
Hearing loss/deafness in years	*8. Postoperative audiometry*
Hearing aid use in the ear to be treated	Time after CI surgery
Treatment contralateral ear	Pure tone audiogram; speech test; sentence test
Type and cause of hearing impairment	*9. Hearing/speech development children*
*4. Implant*	Use of alternative communication
Implantation date	Auditory perception development
Implant manufacturer	Communicative development
Implant type	Progress of hearing/speech development
Implant serial number	Sensory-specific promotion
Explantation (date, reason)	Pedagogical institution/school
*5. Surgery*	*10. Quality of life*
Surgery date and reason	Quality of life questionnaire
Electrode insertion/insertion depth	
Intra-OP functional control	
Carrying out radiological position control of electrode	
Revision surgery	

The complete listing of the data collected can be found in the CI white paper of the DGHNO-KHC [3] and as supplement material: Data blocks CI registry.

*DCIR* German Cochlear Implant Registry, *ABR* auditory brainstem response, *ASSR* auditory steady-state response, *OAE* otoacoustic emissions, *CI* cochlear implant

### Time of data collection

**Fig. 4:**
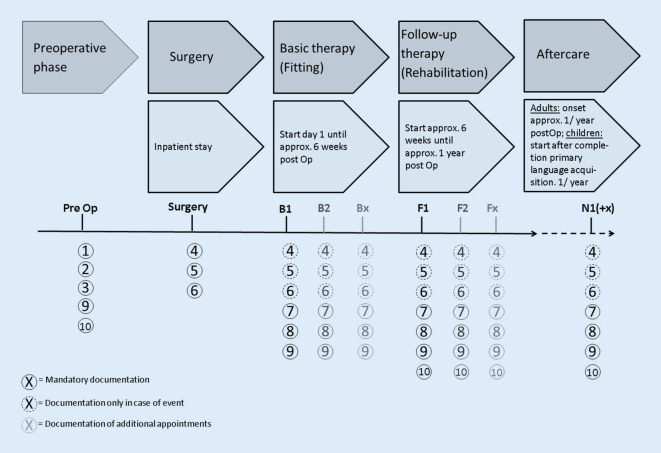
Representation of the time points of data collection in the CI registry. See text for details. *PreOp* preoperative phase, *OP* operative phase, *B* basic therapy, *F* follow-up therapy, and *N* aftercare. *Numbers* *1–10* data blocks to be collected in each phase; *solid circles* mandatory documentation; *dashed circles* incident documentation. *Numbers* given for the respective phase of care (e.g., *B1*, *B2*, *BX*) describe the first, second, or xth contact of the hospital with the patient during the respective phase

The time for documentation of the individual data blocks is also based on the treatment process consented in the CI guideline and the CI white paper, which is divided into five phases: preoperative phase, operative phase, basic therapy, follow-up therapy, aftercare (Fig. 4). Although there are numerous individual, hospital-specific treatment concepts that vary the temporal scope of the individual phases, there is nevertheless scientific consensus on the basic temporal allocation of these stages (Fig. 4). The DCIR therefore envisions documenting at least one time point for data collection for each of the individual phases in order to map all phases of hearing rehabilitation with CI. Since individual phases, e.g., follow-up therapy, may have a different number of individual appointments depending on the patient, the number of data entries may vary significantly.

In principle, the DCIR allows any number of data entry points to be documented for each supply phase. However, at least one entry must be made for each individual phase. As an orientation of time, for the care phases in adults, basic therapy can be assumed up to approx. 6 weeks postoperatively, for follow-up therapy up to approx. 1 year postoperatively, and for aftercare starting approx. 1 year postoperatively. However, individually deviating periods are possible. For children, the time periods are also significantly different. The main features of the data collection periods have already been described in the current version of the CI white paper of the DGHNO-KHC [3].

The structure of the DCIR provides for minimum documentation for individual data blocks in each phase of the care process. By contrast, other data blocks (e.g., data block 6: complications) are only documented in the event of an incident. This approach facilitates a practicable way between documentation scope and feasible effort for the participating hospital. An overview of the mandatory and incident documentation can be found in Fig. 4, which also provides an indication of the time periods for each phase.

### Data evaluation and preparation of annual reports

**Fig. 5:**
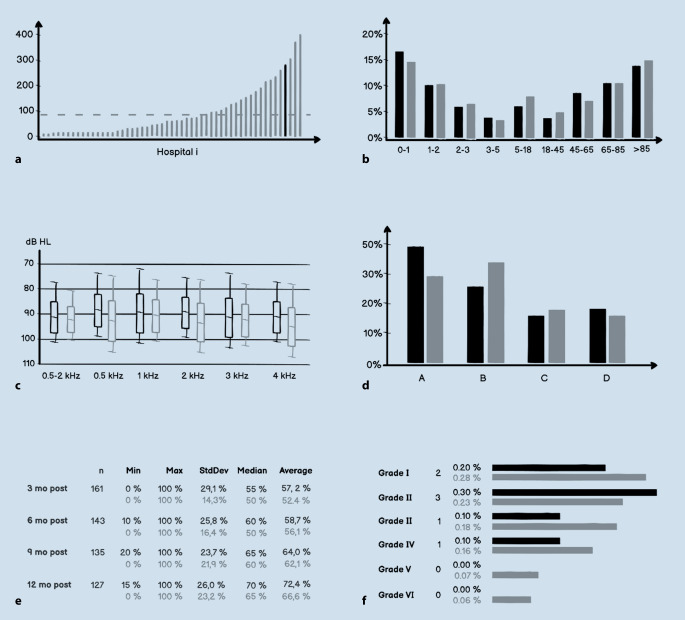
Schematic representation of the evaluation options of the registry data. Fictitious data of a single hospital (*black*) compared to fictitious national key figures (*light gray*) are shown. The example graphs present the annual reports generated for the respective hospital. **a** Presentation of the number of implants of the respective hospital compared to the other hospitals. **b** Presentation of the age distribution of implanted patients of a hospital compared to national data. **c** Presentation of the hospital’s preoperative pure tone audiometric data compared to national data. **d** Distribution of each implant brand (manufacturer) for that hospital compared to national data. **e** Postoperative speech test results (*Freiburger Einsilbertest*, Freiburg Monosyllabic Test) of the hospital at different time points compared to the national data. *mo post* months postoperatively, *Min* minimum, *Max* maximum, *StDev* standard deviation. **f** Recording of facial paresis (grade 1 to 6 according to House–Brackmann) of the respective hospital compared to the national data

The registry operator creates an anonymized annual report for each participating hospital based on the data entered by the hospital (Fig. 2). These data are presented in comparison to the overall national data, thus enabling a relative comparison (benchmarking) for the respective hospital. A hospital’s own data can therefore be viewed in comparison to the average data for Germany as a whole. An exemplary presentation of excerpts from a hospital-specific annual report is shown in Fig. 5.

The registry operator additionally provides the DGHNO-KHC Executive Committee with an anonymized annual report on all data entered into the registry. Identification of individual patients or individual hospitals in relation to the data provided is not possible in the national annual report. Hospitals are only listed anonymously here, so that only anonymized hospital comparisons are possible. Only the registry operator is aware of the identity of the hospital in order to provide feedback to the facility in the event of serious anomalies.

### Development of a logo for the DCIR

**Fig. 6:**
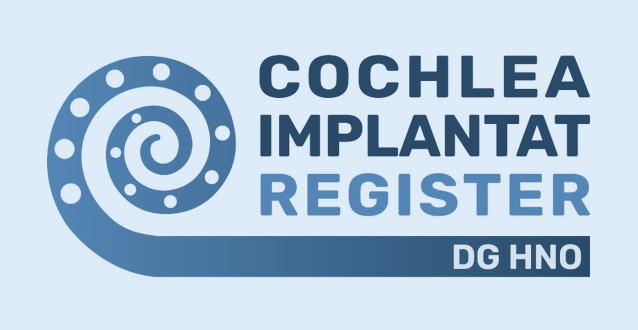
Representation of the logo of the German Cochlear Implant Registry (DCIR)

To enable recognizability of the data and publications collected on the basis of the DCIR, a registry logo was developed in cooperation between the Executive Committee of the DGHNO-KHC and the registry operator, which will be made available to all participating partners of the DCIR for internal and external communication (Fig. 6).

## Results

### Set-up and operation of the DCIR

The practical operation of the DCIR with browser-based data entry was started in January 2022. Since January 2022, pseudonymized data can be entered into the DCIR. In the first 15 months of registry operation, more than 2500 CIs from more than 2000 patients were already successfully entered in the DCIR. It should be noted that the number of implants does not correspond to the number of patients, as patients may also have bilateral CIs. Currently, detailed data analysis is in progress, so that a content-related presentation of the collected results will take place in a separate scientific evaluation (manuscript in preparation).

### Automated data export

The three different mechanisms for data acquisition (Fig. 3) could be implemented in the meantime. The API has been available since fall 2022. This made it possible to successfully find individual solutions for the participating hospitals in order to ensure registry participation.

### Participating hospitals

After completion of the service catalog and the subsequent start of the project, hospitals have been able to declare their willingness to participate in the registry since July 2021. In 2021, 11 hospitals signed a contract to participate in the DCIR, and in 2022, 64 hospitals. By March 2023, a total of 75 hospitals had contractually declared their participation in the DCIR.

### Annual reports 2022

Data entry into the DCIR for the year 2022 was successfully completed for the participating hospitals. At the time of writing, the evaluation process was taking place, and thus the completion of the annual reports for 2022 for the participating hospitals and the preparation of the national annual report for 2022 are anticipated. An evaluation and scientific publication of the content of the national annual report is currently being prepared by the Executive Committee of the DGHNO-KHC.

### Public availability of information on the DCIR

The technical implementation of the DCIR was accompanied by the public provision of information on the objectives and content of the registry. For this purpose, the operator of the registry set up a website with free access (https://www.ci-register.de/).

## Discussion

The development, structuring, and operation of a national clinical registry is a complex task that requires considerable time and financial resources. All of the development steps for the successful operation of the DCIR were realized by the DGHNO-KHC and the registry operator on their own initiative. In addition to the definition of the registry content, the legal and contractual basis for the establishment and operation as well as the development of annual reports and a logo were successfully elaborated. The productive operation of the DCIR started at the beginning of 2022. Since the start of operations, more than 2500 implants from more than 2000 patients have been included in the DCIR in just over 1 year. It is clear that within a short period the data basis of the DCIR will have a very broad foundation to answer scientific questions as well as to derive future quality standards from it. The knowledge gained from the DCIR will thus make a significant contribution to quality control and the further development of scientifically based quality standards in CI care in the future.

In principle, participation in the DCIR is possible for all hospitals that agree to the contractual requirements with the registry operator and fulfill the technical requirements for data entry. In the short time since the DCIR went live, 75 hospitals have already contractually agreed to participate in the CI registry. This is an impressive result, which illustrates the great interest of the hospitals in participating in the registry. In return, participating hospitals receive an annual report that enables benchmarking of hospital data against national averages. This report can also be used directly as a quality report for the hospital. A participating hospital thus not only makes an important active contribution to the further development of new quality standards, but also receives an immediate benefit in the form of a standardized and professional quality report. A CI quality report is also required by the current CI guideline and the CI white paper in order to make a transparent indication of the quality of care of an institution easily available to patients [2, 3].

Providing the technical requirements for data transfer from a hospital presented a particular challenge in the implementation of the DCIR. Thus, in order to achieve the highest possible participation in the DCIR, various technical solutions had to be developed. This included the option of browser-based individual data entry. There are no additional costs to the hospitals from the DCIR for using the API to submit data from local databases. The API functionality is already included in the basic features of the registry. However, a hospital may incur expenses to implement (e.g., programming) the export from the local database. Some hospitals have taken advantage of the data export possibility on the occasion of their wish to participate in the DCIR and have adapted their own database or implemented a new local database, e.g., ENTstatistics (INNOFORCE, Ruggell, Liechtenstein). The registry operator offered the hospitals individual solutions tailored to their specific needs.

Already in the first year after the establishment of the registry, successful data exports of individual participating hospitals could be carried out in this way. Since both the financial resources and the investment possibilities diverge considerably between the hospitals, this aspect was taken into account in the conceptual design of the registry with different access options. This concept allowed hospitals to decide for themselves which level of automation of data transfer (browser-based input, transfer from existing database, or database to be newly set up) they would like to implement. The experience gained in the first year proved the basic usability of all three data entry options presented for the DCIR.

### Assessment of the quality of care

A particular value of a clinical registry lies in the long-term assessment of the quality of care. This includes not only acute complications, but also surveys the long-term quality of outcome of a therapeutic procedure. The scandal surrounding the defective breast implants [11] in particular impressively illustrates this aspect. As a consequence, the IRegG [7] in the meantime has created the legal basis in Germany for the mandatory documentation of a large number of medical implants in a register. The CI is also explicitly mentioned in this law, so that in future not only a voluntary and purely scientifically oriented documentation of CI care will take place, but documentation will be mandatory by law. At present, it remains unclear why other implantable hearing systems, such as active middle ear implants, were not included in the IRegG in addition to CI. Although the exact timing of the start of mandatory documentation of CIs is currently not known, the DGHNO-KHC has already set the professional standard with the registry initiative presented here and has also started a dialogue with the state registry authorities (BfArM). Through the establishment and operation of the DCIR, subject-specific parameters were defined at an early stage and their implementation has been in practice since the beginning of 2022. This initiative can therefore be considered exemplary for other medical implants or even other medical societies.

In addition, a new EU regulation for medical devices came into force in 2021, which requires implant manufacturers to provide, among other things, clinical performance data of their implants in a long-term follow-up. This regulation, known as the Medical Device Regulation (MDR; [12]), poses significant challenges for hospitals and implant manufacturers. It remains to be seen whether the DCIR can also make a contribution with regard to the MDR.

### Acceptance

The CI-provision facilities (CIVE) that demonstrate the necessary structural quality, process quality, and outcome quality on the basis of the CI guideline have been able to obtain a certification in a structured process since 2021 [7]. Across Germany, 47 hospitals have already been awarded the CIVE certificate [13]. A prerequisite for successful certification is the commitment of the hospitals to actively participate in the DCIR. Without a corresponding commitment, the CIVE certificate cannot be awarded to a hospital. Looking at the number of hospitals that committed to participate in the DCIR alone (75 hospitals), there is a significant difference here between certified CI-provision institutions and hospitals participating exclusively in the registry. This difference could be explained from different perspectives. On the one hand, hospitals that may not yet fulfill all conditions for successful certification at the present time could nevertheless have decided to already participate in the registry. It is possible that these hospitals will apply for CIVE certification at a later date. Another explanation could be that hospitals do not seek certification but want to take advantage of registry participation. Since a structured annual report represents a benefit for these hospitals, the DCIR could also be used as a pure database for these hospitals, as they also receive an annual report and the pseudonymized raw data they entered into the registry at the end of the year. In this respect, there are multiple benefits for hospitals from participating in the registry, which already exist from the first year of registry participation.

Another explanation for the high number of hospitals participating in the registry could be the pricing of the registry operator. In the first months of joining the registry, the offer was particularly attractive in financial terms.

Since the start-up of the DCIR, 75 hospitals have already contractually agreed to participate in the registry. This is a very positive development for such a short period, especially considering the number of available hospitals that could participate in the registry. The exact number of institutions offering CI care is not known for Germany. In a survey conducted in 2020 by the DGHNO-KHC in collaboration with the patient self-help group (*Deutsche Cochlea-Implantat Gesellschaft*, DCIG), 70 of 170 ENT hospitals existing in Germany stated that they perform CI treatment. It must be noted that the number of hospitals participating in this survey was not complete, so that the authors assume a number of approximately 100 hospitals offering CI care [14]. Against this background, the number of hospitals (i.e., 75) that agreed to participate in the registry is even more impressive, as it obviously already corresponds to the majority of CI hospitals in Germany. This high number of participants not only demonstrates the great interest of hospitals to actively participate in the registry, but also shows the representative coverage of the clinical data available in Germany by the DCIR that can be expected in the future.

### Comparison with other registries

In Germany, a large number of clinical registries have already been introduced very successfully in the past to collect care parameters. One example is the Trauma Registry of the Academy of Trauma Surgery (AUC; [5]). This registry has been in operation since 1993 and can be regarded as pioneering for medical–scientific registries that are primarily aimed at improving the quality of care. The objective of the CI registry is therefore similar to already established concepts, since the scientific approach, supported by the medical society (DGHNO-KHC), is not motivated by commercial interests, but by medical, scientific, and quality-oriented interests. With regard to the collected data, it was therefore out of the question that only anonymized data analysis would be performed. On the other hand, in order to enable realistic benchmarking, it must be possible to compare the respective hospital data with the national average data. The evaluation system presented here combines both the hospital interests (anonymous benchmarking) with the interests of the national medical society to create a national annual report. For the first time, a very large number of care processes and patients cared for is collected nationwide by this approach.

### International comparison

As a comparison internationally, some CI registries already exist. The Swiss and the French CI registry can be mentioned as examples [15, 16]. In Switzerland, the registry was introduced as early as 1992 and has been operated continuously since then. In a recent paper [17] it was shown for Europe that in 2021, CI registries were established in only four countries (approx. 10% of the countries in Europe). In this respect, the introduction of the DCIR is not the first approach to quality control of CI care in a European country. However, taking into account the parameters collected in other European CI registries, the approach of the DCIR presented here to collect relevant parameters over ten defined data blocks seems unique and innovative. The main differences between the DCIR and other registries are the recording of the entire CI care process and the lifelong follow-up, which are important quality parameters.

### Limitations

Despite a very positive development of the DCIR so far, the methodological approach is, nevertheless, subject to some limitations, which will be discussed in the following.

The main challenge in securing the long-term operation of the registry is to collect as complete data as possible from CI patients. Currently, participation in the registry is purely voluntary for hospitals, motivated by the desire to further improve the quality of CI care, the future development of new quality standards, and also the provision of an annual report for participating hospitals. There is currently no obligation to provide the data. As a point of reference for assessing the coverage of available data by the registry, the number of surgeries performed can be used on the basis of the DRG statistics provided by the Federal Statistical Office. According to this, 4359 CI operations [18] took place in Germany in 2021. Considering the number of implants included in the DCIR in about 15 months (as of March 2023: > 2500 implants), the penetration of the registry for CI care in Germany seems to be very high even at this point in time: Approximately 50% of the implants have already been included in the registry during the short operating time of the DCIR, assuming approximately the same number of cases of care in 2021 and 2022. Even though this is a remarkable success in the initial phase of the registry, it must be critically noted that approx. 50% of the implantations are currently not yet documented. Whether this is due to individual technical difficulties that have not yet been resolved, incomplete data transfer, or hospitals that are not willing to participate cannot be conclusively assessed at this time. At the latest with the future implementation of the IRegG and its application to CI, a complete, since it is obligatory, documentation of all implantations is foreseeable. The DCIR is also carrying out important technical preliminary work in this area.

Ensuring data quality will be essential for the scientific usability of the registry. This concerns both the completeness of the provided data sets and, in particular, the content plausibility check of the recorded data. Thus, the quality control of the DCIR will also be of great importance in the future.

At present, data transfer to the registry requires informed consent to be obtained from the respective patient. This is a time-consuming process and there is also the possibility that a patient does not give their consent to the data transfer. Currently, the administrative burden lies with the participating hospitals. It is to be hoped that patients with CI implants will continue to give their consent to this, also on the basis of the expected results and the long-term influence on future quality standards, in order to support the DCIR. An appropriate presentation of the DCIR and the resulting data should therefore also be made available to CI patients in order to achieve appropriate support from both the patients and the patient self-help organizations. With the future introduction of the IRegG [7], the documentation of the collected data will then become legally mandatory and will no longer require active consent from a patient.

The structure of the CI registry is based on the content of the German CI guideline and the CI white paper. In principle, the care process presented here can be applied to adults and children alike. However, it is obvious that in addition to a high degree of congruence of the parameters to be collected (e.g., technical data or demographic data), age-specific parameters must also be gathered for a large number of the variables collected. Especially with regard to the assessment of success, a multitude of challenges arise here that make direct comparability of child and adult data difficult. Currently, the registry structure is consistent with the consensus content of the CI guideline and CI white paper. It remains to be seen whether a change in data blocks or data fields will be required with the future update of the CI guideline or CI white paper.

### Costs and refinancing

Further development of the quality of CI care expected from the registry is not only in the interest of patients and hospitals, but also in the direct interest of health costs payers, e.g., health insurances. Also in the future, recurring costs for the operation of the registry are to be expected from the participating hospitals as well as from the DGHNO-KHC. It is therefore obvious that the described initiative must find the support of the health costs payers in order to ensure the long-term operation of the DCIR. This is of particular importance, since only the long-term follow-up of implant safety, of possible complications, but also of the outcome quality of CI care can offer enormous potential for scientifically based quality control. The development of the basic principles, the structuring, and the introduction of the DCIR up to its successful start-up has been realized exclusively by the own initiative of the DGHNO-KHC as well as the participating hospitals. The financial investments required for this on the part of the DGHNO-KHC represent a considerable financial burden. Hospitals also bear annual costs for participation in the registry. Therefore, the DCIR represents a relevant financial investment both for the national medical society and for the hospitals. There is no question that the refinancing of the registry, or its long-term operation, requires the financial support of the health costs payers.

## Practical conclusion

- The work presented here describes the structuring, development, and successful establishment of the German Cochlear Implant Registry (DCIR).
- By implementing the preliminary work done in the national CI guideline and the CI white paper regarding the parameters relevant to the structure, process, and outcome quality, a consistent transfer of this content to the DCIR was achieved.
- After the introduction of certification for CI-provision institutions , the introduction of the DCIR represents another essential milestone for the future science-based quality control of CI care in Germany.
- The initiative of the German Society of Otorhinolaryngology, Head and Neck Surgery (DGHNO-KHC) described here, supported by the participating hospitals, provides active quality assurance in the interest of the patients and at the same time scientific work.
- The registry can therefore be considered exemplary for other areas of medical care and thus also sets internationally visible standards.

## Supplementary Information

Data blocks of the DCIR

## Declarations

For this article no studies with human participants or animals were performed by any of the authors. All studies mentioned were in accordance with the ethical standards indicated in each case.

The supplement containing this article is not sponsored by industry.
