# Supplementary material for: Structure and establishment of the German Cochlear Implant Registry (DCIR)
Source: HNO. 2023 Jul 18;71(Suppl 1):82–92. doi: 10.1007/s00106-023-01310-0 (PMC10409674; doi:10.1007/s00106-023-01310-0)
Supplement: Supplementary file 1 — Data blocks of the DCIR [file 106_2023_1310_MOESM1_ESM.docx]

**Data blocks CI register**

***1-3: Data blocks created once***

**1. Basic data**

ID (code) Providing facility (hospital):

Patient ID Pseudonym:

Date of birth: DD.MM.YYYY

Date of death: DD.MM.YYYY

Gender: W/M/D/ka

Mother tongue German: yes/no/not collected

**2. Preoperative audiometry (to be created for each side)**

Page: R/L

Pure tone audiogram LL/KL (125/250/500/750/1000/2000/4000/8000Hz): 0-120 dB HL / not determinable

Language test:

Freiburger, hearing loss numbers (KH without HG, 50%, 100 dB SPL if below 50%): 0-100 dB SPL / not collected

Freiburger Einsilber (KH without HG 65 dB SPL): 0-100 % / not collected

Freiburger Einsilber mEV (KH without HG, min. 95 dB SPL): 0-100 % / not surveyed

Freiburger Einsilber (FF, with HG 65 dB SPL): 0-100 % / not surveyed

Mainz Children's Speech Test (KH, without HG, I/II/III, 65 dB SPL): 0-100% / not collected

Mainz Children's Language Test (FF, with HG, I/II/III, 65 dB SPL): 0-100% / not collected

Göttingen Speech Intelligibility Test (KH, without HG, I/II, 65 dB SPL): 0-100% /not collected

Göttingen Speech Intelligibility Test (FF, with HG, I/II, 65 dB SPL): 0-100% /not collected

Rhyme test OlKi at rest (KH, without HG, 65 dB SPL): 0-100% / not collected

Rhyme test OlKi at rest (FF, with HG, 65 dB SPL): 0-100% / not collected

Sentence tests (FF, S0N0) with HG, speech level 65 dB SPL (single selection)

OlSa at rest: 0-100% / not collected

OlSa threshold in noise (SRT50): -30 to +30 dB SNR /not collected

GöSa at rest: 0-100% / not collected

GöSa threshold in noise (SRT50): -30 to +30 dB SNR /not surveyed

HSM at rest: 0-100%/not collected

HSM Noise: 0-100 %/not collected

HSM SNR: -5, 0, +5, +10 dB/not raised

OlKiSa at rest: 0-100% / not collected

OlKiSa in noise (SRT50): -30 to +30 dB /not collected

Objective measurements

OAE (TEOAE/DPOAE) detectable: yes / no / not collected

ABR non-freq. specific (LL/KL): no stimulus response / threshold in dB nHL / not surveyed.

ABR/ASSR frequency-specific (500/4000Hz): no stimulus response / 0-120 dB HL / not elicited.

**3. Preoperative hearing history (to be taken for each side)**

Page: R/L

Time of hearing loss: prelingual / perilingual / postlingual / not collected

Hearing loss (CI ear) in years: 0-1, 1-5, 5-10, 10-20, >20, not collected

Deafness (CI ear) in years: 0-1, 1-5, 5-10, 10-20, >20, not collected.

Current hearing aid use in the ear to be fitted: yes / no

Care of the opposite ear: normal hearing, hearing loss (no hearing loss, HG, KL-HG, IHG, CI, other, not surveyed)

Cause of hearing impairment: known: yes / no / not raised (if yes, multiple selection possible): genetic, age-related, syndromal, traumatic, endocrinological, toxic, infectious, atypical anatomy inner ear, atypical anatomy auditory nerve, atypical anatomy central auditory pathway, Meniere's disease, vestibular neurinoma, other)

Type of hearing impairment: cochlear / neural / other / not ascertained

***4-10: Data blocks created multiple times***

**4. Implant (to be created for each side/intervention)**

Page: R/L

Implantation date: DD.MM.YYYY

Implant manufacturers (1: Advanced B., 2: Cochlear, 3: MedEl, 4: Oticon, 5: Other): 1-5

Implant designation: <list, other>

Implant serial number:

Explantation: y/n

Explantation date: DD.MM.YYYY

Reason for explantation according to classification: (B1, B2, C, D, )

**5. Operation (to be created for each page)**

Page: R/L

Operation date: DD.MM.YYYY

Reason for surgery: 1: Primary surgery / 2: Revision surgery: 1-2

Electrode insertion (1: round window / 2: cochleostomy / 3: other): 1-3

Insertion depth (1: partial / 2: complete): 1-2

- Intra-OP functional checks (electrode impedance and/or E-CAP): regular / not regular / not performed

Perform radiological position control Electrode: yes / no

if yes (1: regular / 2: not regular / 3: N/A); and if yes, which method? (1: conventional X-ray/2: DVT/3: CT/4: other): 1-4

Revision operation: Y/N

Indication revision surgery: medical, technical, other, n.d.

Classification implant function: A-D

Implant status during revision surgery 1: Leave device / 2: Change / 3: Explantation: 1-3

If reimplantation: 1,2...n-th implant change

**6. CI-related complications**

Page: R/L

Electrode misalignment in need of revision: y/n

Facial paresis: House-Brackmann grade I-VI

Inpatient admission was required due to CI-related complications: y/n

Meningitis after CI fitting: y/n

Death associated with CI fitting: y/n

**7. CI use and rehabilitation progress.**

Page: R/L

Implant function (classification according to Consensus 2005): A, B1, B2, C, E, not surveyed

CI usage time (time in hrs/day): 0-24, kA

Duration of use collected by (1: patient/parent statement /2: data logging/ 3: n/a): 1-3

Current rehabilitation status:
process phase (1: basic therapy /2: follow-up therapy / 3: aftercare / 4: unknown / 5: not collected): 1-5.

**8. Postoperative audiometry**

Page: R/L

Time after CI surgery in months: 0-XX (Automatic)

Pure tone audiogram (unpowered) LL/KL (125/250/500/750/1000/2000/4000/8000Hz): 0-120 dB / not determinable

Speech test (with CI, FF, speech level 65 dB SPL):

Freiburg figures: 0-100 %

Freiburg monosilver: 0-100 %

Mainz Children's Language Test (I/II/III): 0-100% / not collected

Göttingen Speech Comprehension Test (I/II): 0-100% /not collected

Rhyme test OlKi: 0-100% / not collected

Sentence tests (FF, S0N0) with CI, speech level 65 dB SPL (single selection)

OlSa at rest: 0-100% / not collected

OlSa threshold in noise (SRT50): -30 to +30 dB SNR / not surveyed

GöSa at rest: 0-100% / not collected

GöSa threshold in noise (SRT50): -30 to +30 dB SNR / not surveyed

HSM at rest: 0-100%/not collected

HSM Noise: 0-100 %/not collected

HSM SNR: -5, 0, +5, +10 dB/not raised

OlKiSa at rest: 0-100% / not collected

OlKiSa in noise (SRT50): -30 to +30 dB / not surveyed

**9. Hearing/language development (children)**

Use of alternative communication methods (e.g. DGS, GUK, Talker): yes / no / not collected

Auditory perception development (e.g. parent questionnaire): inconspicuous / conspicuous / not surveyed

Communicative development (e.g. parent questionnaire): inconspicuous / conspicuous / not surveyed

Progress of hearing/language development: regular/not regular/not collected

Status of speech comprehension: inconspicuous / conspicuous / not surveyed

Status phonetics-phonology: inconspicuous / conspicuous / not surveyed

Status lexicon semantics: inconspicuous / conspicuous / not raised

Status syntax morphology: inconspicuous / conspicuous / not surveyed

Status communication pragmatics: inconspicuous / conspicuous / not surveyed

Sensory-specific promotion / preventive measure is currently being carried out: yes / no / unknown/ not surveyed

Pedagogical institution/school: regular kindergarten / regular school / other institutions / not surveyed

**10. Quality of life**

Quality of life questionnaire collected: NCIQ/ other / not collected

Elementary sound perception NCIQ1 (0-100)

Speech and music perception NCIQ2 (0-100)

Control of own voice NCIQ3 (0-100)

Psychosocial consequences NCIQ4 (0-100)

Activity behavior NCIQ5 (0-100)

Social contacts NCIQ6 (0-100)

NIIQ Total score NCIQTotal (0-100)
